# Direct Regulation of *CLOCK* Expression by REV-ERB

**DOI:** 10.1371/journal.pone.0017290

**Published:** 2011-03-29

**Authors:** Christine Crumbley, Thomas P. Burris

**Affiliations:** The Scripps Research Institute, Jupiter, Florida, United States of America; New Mexico State University, United States of America

## Abstract

Circadian rhythms are regulated at the cellular level by transcriptional feedback loops leading to oscillations in expression of key proteins including CLOCK, BMAL1, PERIOD (PER), and CRYPTOCHROME (CRY). The CLOCK and BMAL1 proteins are members of the bHLH class of transcription factors and form a heterodimer that regulates the expression of the *PER* and *CRY* genes. The nuclear receptor REV-ERBα plays a key role in regulation of oscillations in *BMAL1* expression by directly binding to the *BMAL1* promoter and suppressing its expression at certain times of day when REV-ERBα expression levels are elevated. We recently demonstrated that REV-ERBα also regulates the expression of NPAS2, a heterodimer partner of BMAL1. Here, we show that REV-ERBα also regulates the expression another heterodimer partner of BMAL1, CLOCK. We identified a REV-ERBα binding site within the 1^st^ intron of the *CLOCK* gene using a chromatin immunoprecipitation – microarray screen. Suppression of REV-ERBα expression resulted in elevated *CLOCK* mRNA expression consistent with REV-ERBα's role as a transcriptional repressor. A REV-ERB response element (RevRE) was identified within this region of the *CLOCK* gene and was conserved between humans and mice. Additionally, the *CLOCK* RevRE conferred REV-ERB responsiveness to a heterologous reporter gene. Our data suggests that REV-ERBα plays a dual role in regulation of the activity of the BMAL1/CLOCK heterodimer by regulation of expression of both the *BMAL1* and *CLOCK* genes.

## Introduction

Circadian rhythms play an essential role in coordinating the timing of various physiological processes. In mammals, the master clock for circadian rhythm is maintained in the suprachasmatic nucleus (SCN) in the brain, but many peripheral organs maintain semi-autonomous clocks that can be set using signals from the SCN or other signals, such as nutrient status. The circadian clock is regulated by a transcriptional/translational feedback loop that involves several key proteins including circadian locomotor output kaput (CLOCK). *CLOCK* was initially discovered in a mutagenesis screen for altered circadian phenotypes in mice [1]. The *CLOCK* mutant mice had an increased period of circadian activity, as determined by wheel running experiments, and became arrhythmic in constant darkness. The *Clock* gene was positionally cloned revealing its identity as a member of the basic helix-loop-helix family of transcription factors [2]. *Clock* is expressed in the SCN of mice as well as in humans, but also displays a wider pattern of expression that includes the liver where it may play a role in regulation of the circadian rhythm in this tissue [3], [4], [5].

CLOCK functions as a heterodimer with another bHLH transcription factor, the Brain and Muscle ARNT (Aryl hydrocarbon receptor nuclear translocator)-like 1 (BMAL1). The CLOCK/BMAL1 heterodimer binds to E-box elements in the promoters of the *PERIOD* (*PER*) and *CRYTOCHROME* (*CRY*) genes increasing their transcription. The PER and CRY proteins also heterodimerize and inhibit CLOCK/BMAL1 activity leading to reduction in PER and CRY expression, thereby completing the core feedback loop responsible for the circadian rhythm. Two nuclear receptors that are expressed in a circadian pattern modulate the activity of this core loop. The retinoic acid receptor-related orphan receptor α (RORα) and REV-ERBα directly regulate the expression of *BMAL1* by binding to a specific ROR/REV-ERB response element in the *BMAL1* promoter [6]. RORα stimulates BMAL1 whereas REV-ERBα represses transcription. Studies using genetically modified mice show that RORα and REV-ERBα play a role in maintaining circadian rhythm. The RORα staggerer mutant mice (RORα^sg/sg^) and RORα^−/−^ mice have shortened circadian periods [7]. The REV-ERBα null mice also have a shortened circadian period and greater light-induced phase responsiveness [8]. Given that RORα and REV-ERBα have been shown to regulate *BMAL1* expression, we investigated whether another BMAL1 heterodimer partner, NPAS2, was regulated by these 2 nuclear receptors and found that it was a direct target gene [9]. Thus, we next sought to determine if RORα and/or REV-ERBα might regulate the expression of *CLOCK*.

## Materials and Methods

### ChIP-on-chip

HepG2 cells (ATCC, Manassas, VA) were infected with REV-ERBα adenovirus and harvested for use in ChIP/microarray as previously described by our laboratory [9], [10], [11], [12].

### Chromatin Immunoprecipitation (ChIP)

HepG2 cells were transfected with 50 nM control or REV-ERBα siRNA (Dharmacon) using Lipofectamine RNAiMax (Invitrogen) according to manufacturer's instructions. Media was changed 24 hrs after transfection. Cells were fixed using formaldehyde 72 hrs after transfection. The ChIP-IT Express kit from Active Motif was used. Cells were lysed and then sonicated to shear the chromatin. Immunoprecipitations were incubated overnight at 4°C. The ChIP reactions contained 5 µg of the following antibodies: IgG (Active Motif), anti-RNA Pol II (Active Motif), anti-hREV-ERBα (Cell Signaling), and anti-hNCoR (Santa Cruz sc-8994). The ChIP reactions were washed and chromatin was eluted, according to manufacturer's instructions. PCR reactions were performed using 50 µL PCR Supermix High Fidelty (Invitrogen), 1.5 µL of each primer (10 µM), and 10 µL of eluted chromatin. The IgG and anti-RNA Pol II were provided in the ChIP-IT human control kit (Active Motif). The thermocycler program was 94°C for 3 mins; 40 cycles of 94°C for 20 s, 65°C for 30 s, 72°C for 30 s; 72°C for 2 min. PCR products were visualized using ethidium bromide gel electrophoresis.

### Electrophoretic Mobility Shift Assay (EMSA)

The REV-ERBα coding sequence was excised from p3xFLAG-REV-ERBα using BamHI and HindIII. The vector pcDNA3.1+ (Invitrogen) was digested with BamHI and HindIII. All fragments were gel purified, then ligated using T4 DNA Ligase (Promega). The constructs were verified by sequencing. The constructs contain a T7 promoter for in vitro transcription and translation and were used to generate protein for EMSA using the TNT T7 kit (Promega). The putative *CLOCK* RevRE and the *BMAL1* RevRE were annealed and labeled with α^32^P dATP using Klenow polymerase (Promega). Binding reactions contained binding buffer (Promega), labeled probe, and REV-ERBα protein. The resulting complexes were loaded onto 5% TBE gels (Biorad) and analyzed by autoradiography. For competition experiments, unlabled CLOCK RevRE (wt or mt) was added at 10-, 50- or 100-fold molar excess. The sequences of the probes utilized in the EMSA are indicated below:

hCLOCK_ROREwt_F: TTGGAATAAAGTGGGTCACAAGGC
hCLOCK_ROREwt_R: TTGCCTTGTGACCCACTTTATTCC
hCLOCK_ROREmut_F: TTGGAATAAAGTGTTTCACAAGGC
hCLOCK_ROREmut_R: TTGCCTTGTGAAACACTTTATTCC
hBmalI_ROREwt_F: TTGAAGGCAGAAAGTAGGTCAGGGACGGGACGGAG
hBmalI_ROREwt_R: TTCTCCGTCCCTGACCTACTTTCTGCCTTC

### Cell Culture and Cotransfection Assay

The putative *CLOCK* RevRE and a mutated RevRE were synthesized as a three-times repeat (3×RevRE) with XhoI and MluI restriction sites on the ends (IDT). The wild-type and mutant *CLOCK* 3×RORE oligos were digested with XhoI and MluI (Promega). The pTL-Luciferase vector was also digested with XhoI and MluI (Promega). All fragments were gel purified and ligated using T4 DNA Ligase (Promega). The constructs were verified by sequencing. The p3x-FLAG-REV-ERBα has already been described previously [13]. Human HepG2 cells were maintained and propagated in minimum essential medium supplemented with 10% fetal bovine serum at 37°C under 5% CO_2_. For transfections, HepG2 cells were plated in 96-well plates at a density of 15×10^3^ cells/well 24 h prior to transfection. Transfections were performed using Lipofectamine 2000, according to manufacturer's instructions (Invitrogen). Per well, the transfection mixture included 50 ng *Renilla* luciferase as an internal control, 100 ng of the appropriate *CLOCK* luciferase construct, and 100 ng of the p3x-FLAG-REV-ERBα expression construct. The luciferase activity was measured using the Dual-Glo luciferase assay system 24 h after transfection (Promega). The luciferase readings were normalized by well to the Renilla readings. For treatment with GSK4112, HepG2 cells were plated into 12-well plates. Cells were then treated with 10 µM GSK4112 or equivalent volume of vehicle. Treatment lasted for 24 hrs. Cells were harvested for RT-PCR determination of *CLOCK* expression.

### siRNA Transfection

The siRNAs targeting RORα, RORγ, and REV-ERBα were purchased from Dharmacon (Thermo Fischer). The siRNA was transfected into HepG2 cells using Lipofectamine RNAiMax, according to manufacturer's instructions (Invitrogen). The siRNA-treated HepG2 cells were incubated for 24 hours before being harvested for mRNA isolation.

### RT-PCR

Extraction of mRNA, synthesis of cDNA, and quantitative PCR were performed as described previously [14]. The cyclophilin B (M60857) gene was used as the control. All primers were designed for human genes. Primer sequences are listed below.

Cyclophilin B forward: 5′- GGAGATGGCACAGGAGGAAA -3′
Cyclophilin B reverse: 5′- CGTAGTGCTTCAGTTTGAAGTTCTCA -3′
REV-ERBα forward: 5′- TTCCGCTTCGGTGGAGCAGC -3′
REV-ERBα reverse: 5′- CCGGTTCTTCAGCACCAGAG -3′
CLOCK forward: 5′- TGGGAATCCCTCAACTCAAC -3′
CLOCK reverse: 5′- GACTGAGGGAAGGTGCTCTG -3′

### Statistical Analysis

In the co-transfection assays, the values indicated represent the means ± S.E. from eight independently transfected wells. In the RT-PCR assays, the values indicated represent the means ± S.E. from four independently transfected wells. The experiments were repeated at least three times.

## Results

**Figure 1:**
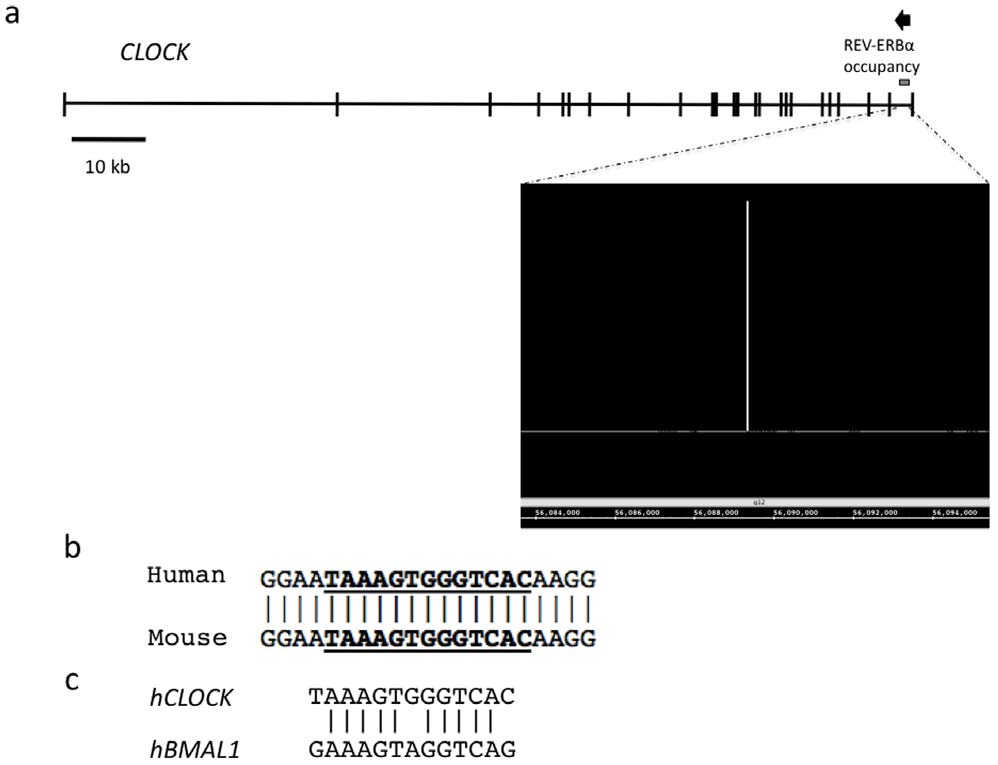
Identification of a REV-ERBα binding site within the *CLOCK* gene. (a) Significant REV-ERBα occupancy was observed within the *CLOCK* gene within the 1st intron. The genomic structure of *CLOCK* is shown with REV-ERBα occupancy illustrated as the gray line. The arrow indicates the direction of transcription. The raw ChIP/chip data is shown in a window beneath the gene as is a screen shot from the integrative genome browser. (b) The region of REV-ERBα occupancy was scanned for conserved RevRE using the Evolutionarily Conserved Region Browser and MatInspector. A RevRE was found to be conserved between mice and humans, the alignment of the RevRE is shown. (c) Alignment of the *CLOCK* RevRE to a characterized RevRE in the *Bmal1* promoter.

A ChIP/chip screen was performed to determine regions where REV-ERBα is bound within the genome as previously described [9]. Significant REV-ERBα occupancy was observed with the *CLOCK* gene, as diagrammed in Figure 1a. Using the Evolutionarily Conserved Region Browser [15], it was determined that a putative RevRE is located within the *CLOCK* gene that is conserved between mice and humans, as shown in Figure 1b. The same site was detected using MatInspector [16]. An alignment between the *CLOCK* RevRE site to a characterized RevRE in the *BMAL1* promoter is shown in Figure 1c.

**Figure 2:**
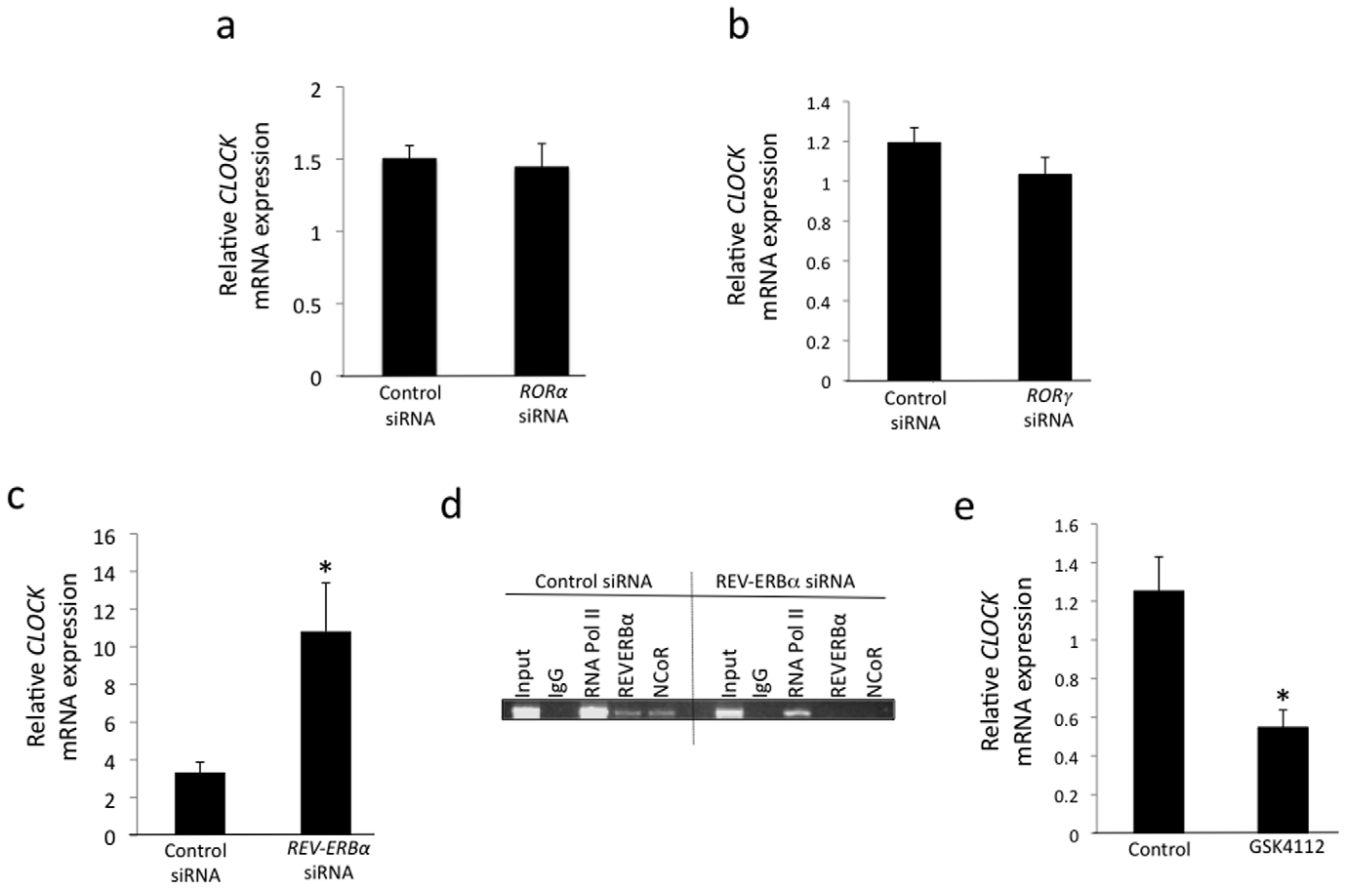
REV-ERBα is required for normal expression of *CLOCK*. HepG2 cells were transfected with siRNAs targeting RORα (78% reduction), RORγ (50% reduction) and REV-ERBα (57% reduction) to reduce their expression. *CLOCK* expression was then examined by RT-PCR. *CLOCK* expression was elevated when REV-ERBα expression was reduced (c), but unaffected by alteration of RORα (a) expression or RORγ (b). Data shown is mean ± SEM where n = 4. (d) ChIP assay illustrating REV-ERBα and NCoR occupancy of the 1^st^ intron of the *CLOCK* gene. (e) The synthetic REV-ERBα agonist GSK4112 suppresses *CLOCK* gene expression in HepG2 cells. *, p<0.05.

We suppressed RORα (including RORα1 and RORα4), RORγ, and REV-ERBα expression in HepG2 cells by transfecting the cells with specific siRNAs. When RORα expression was decreased by ∼78% using siRNA, the expression of *CLOCK* was unchanged (Figure 2a). A similar lack of effect was observed when we suppressed RORγ expression by ∼50% using siRNA (Figure 2b). However, when REV-ERBα expression was decreased by ∼57% using siRNA, the expression of *CLOCK* was increased 3.2-fold, as shown in Figure 2c, indicating that endogenous REV-ERBα exerts a repressive effect on *CLOCK* expression. We confirmed occupancy of REV-ERBα at the site identified by the ChIP-on-chip screen by ChIP and also noted that the corepressor NCoR was also recruited to the site (Figure 2d). When we examined occupancy following knock-down of REV-ERBα expression as indicated above we noticed loss of REV-ERBα occupancy as well as loss of NCoR occupancy (Figure 2d) indicating that NCoR recruitment was mediated by REV-ERBα and consistent with the loss of repressive effects of this receptor and the increase in *CLOCK* gene expression we noted in Figure 2c. We next assessed the ability of a synthetic REV-ERBα agonist to modulate the expression of *CLOCK* expression in HepG2 cells. GSK4112 (SR6452) binds directly to the ligand binding domain of REV-ERBα and increases the recruitment of NCoR leading to increased repression of target genes [17], [18], [19], [20]. As shown in Figure 2e, treatment of the HepG2 cells with GSK4112 results in repression (∼55%) of *CLOCK* gene expression. Interestingly, overexpression of REV-ERBα in HepG2 cells did not result in any change in *CLOCK* gene expression (data not shown), suggesting that the endogenous REV-ERBα levels may be saturating.

**Figure 3:**
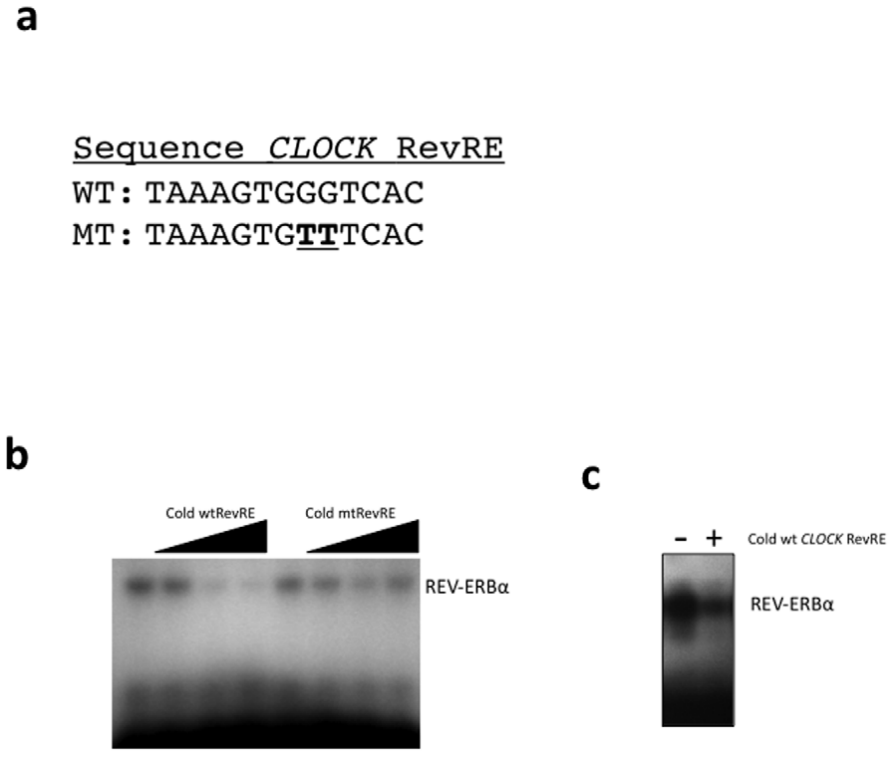
Identification of a functional RevRE within the *CLOCK* gene by EMSA. (a) The sequences of the wild-type *CLOCK* RevRE and mutant *CLOCK* RevRE are shown. (b) Demonstration of direct binding of REV-ERBα to radiolabeled *CLOCK* RevRE DNA. Binding of REV-ERBα to labeled DNA decreased with addition of unlabeled (cold) *CLOCK* RevRE, but not mutant *CLOCK* RevRE. The arrow indicates increases amounts of cold DNA added (10×, 50×, and 100× molar excess). (c) EMSA illustrating that REV-ERBα binds to radiolabeled BMAL1 RevRE and that 100-fold molar excess of CLOCK RevRE DNA can compete for binding to REV-ERBα.

The putative RevRE was examined for REV-ERBα binding using an EMSA. Synthetic oligonucleotides encoding the putative *CLOCK* RevRE were radiolabeled, and then incubated with REV-ERBα protein produced *in vitro*. The sequences of the oligonucleotides are shown in Figure 3a. Direct binding of REV-ERBα to the wild-type *CLOCK* RevRE is shown in Figure 3b. Addition of unlabeled wild-type *CLOCK* RevRE probe was able to displace the radiolabeled *CLOCK* RevRE from REV-ERBα, as shown in Figure 3b while an excess of unlabeled mutant *CLOCK* RevRE was unable to displace the radiolabeled *CLOCK* RevRE probe from the REV-ERBα protein demonstrating specificity. We also assessed the ability of the *CLOCK* RevRE probe to compete with the well-characterized *BMAL1* RevRE. The BMAL1 RevRE was radiolabeled and then 100-fold molar excess of unlabeled CLOCK RevRE was included as a competitor. As illustrated in Figure 3c, the *CLOCK* RevRE was able to compete for binding of REV-ERBα to the *BMAL1* RevRE.

**Figure 4:**
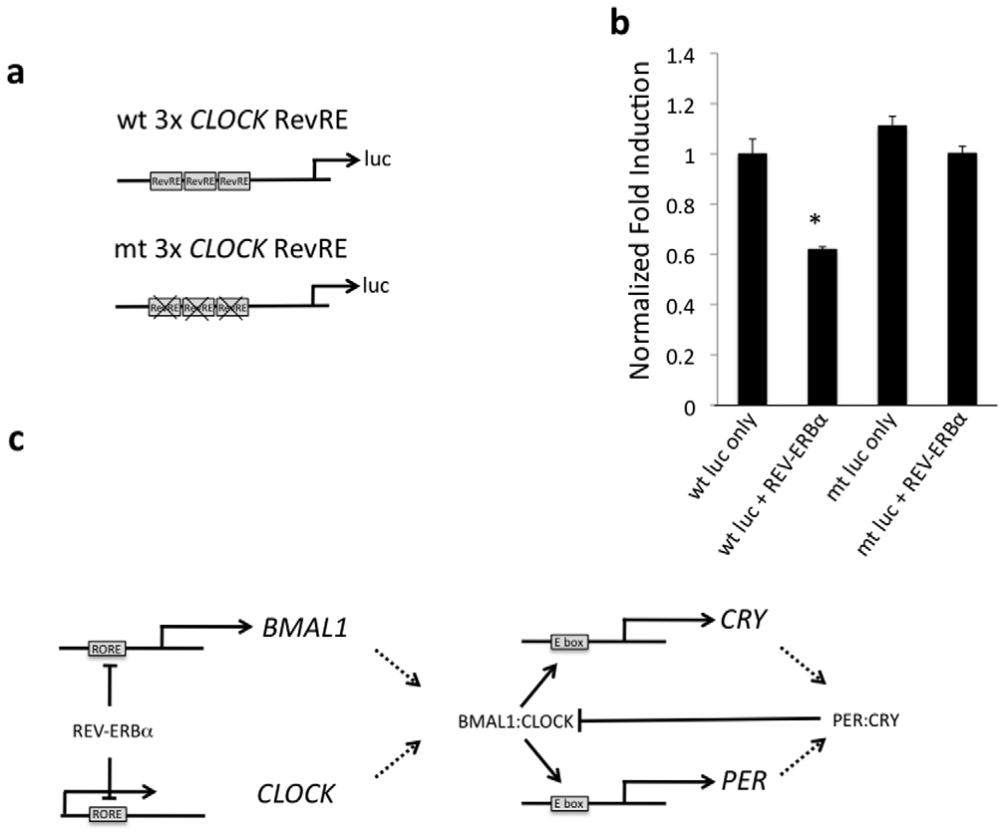
Identification of a functional RevRE within the *CLOCK* gene using reporter-luciferase assays. (a) The reporter constructs contain a three-times repeat of the putative CLOCK RevRE cloned upstream of the firefly luciferase gene. (b) REV-ERBα was co-transfected with a luciferase reporter containing the wild-type 3×RevRE leading to reduced expression of luciferase relative to the reporter alone. The expression of luciferase from the mutant 3×RORE was unaffected by REV-ERBα co-transfection. Data shown is mean ± SEM, n = 8. *, p<0.05. (c) Proposed model for coordinated regulation of *BMAL1*/CLOCK heterodimers.

Due to the location of the putative RevRE within the 1^st^ intron of the *CLOCK* gene, we generated a three-times repeat of the RevRE to clone upstream of the firefly luciferase gene (Figure 4a). When the wild-type 3×RevRE reporter construct was transfected into HepG2 cells, the co-transfection of REV-ERBα suppressed luciferase expression relative to reporter alone (Figure 4b). The expression of luciferase from the mutant 3×RevRE reporter construct was unaffected by co-transfection of REV-ERBα when transfected into HepG2 cells. Similar results were observed in HEK293 cells (data not shown).

## Discussion

CLOCK heterodimerizes with BMAL1 and maintains the circadian expression of target genes containing E-boxes in their promoters. *BMAL1* is a known direct target gene of RORα and REV-ERBα, whose circadian expression influences the expression of *BMAL1*. However, little is known about how the dimerization partner of BMAL1, CLOCK, is regulated at the transcriptional level. We recently demonstrated that another BMAL1 heterodimerization partner, NPAS2, is regulated by RORα and REV-ERBα providing for a potential mechanism for coordinated expression of these two bHLH factors that regulate the circadian rhythm [9]. In this study, we found that another important factor that heterodimerizes with BMAL1, CLOCK, is also a target of REV-ERBα. BMAL1 cannot function independently of a dimer partner such as CLOCK or NPAS2, thus it is logical that both components of the heterodimer may share some regulatory components. Coordination of their regulation by simultaneous modulation by REV-ERBα may be one mechanism that this can occur. However, it is intriguing that RORα regulates *BMAL1* expression but does not appear to play a role in regulation of *CLOCK*. We also noted that *CLOCK* is not responsive to RORγ, indicating that this particular response element in the *CLOCK* gene is selective for REV-ERBα. Given the similarity between the *BMAL1* and *CLOCK* RevREs this is surprising and it is currently unclear what differences may be driving the selectivity. It is possible that the REV-ERBα selectivity for regulating the *CLOCK* gene may be a function of the HepG2 cell line used in this study and in other cell types RORα may play a significant role. Alternatively, other BMAL1 partners such as NPAS2 may play a significant role in maintaining the proper ratio of BMAL1 to heterodimerization partner. It is interesting to note that in the HepG2 cells we previously observed circadian oscillations in the expression of *BMAL1*, *RORα* and *NPAS2* but not *REV-ERBα* following a serum shock [9]. We also observed that *CLOCK* does not oscillate in the HepG2 cells (data not shown), which is consistent with the lack of oscillation in *REV-ERBα* and the lack of responsiveness to the oscillating *RORα*. Again, this pattern of regulation may be specific for HepG2 cells or in cells or tissues where *CLOCK* does not exhibit a strong circadian pattern of expression. Clearly, REV-ERBα plays an important role in the circadian pattern of expression of CLOCK in some circumstances since the REV-ERBα null mouse exhibits a loss in the pattern [8]. Our data suggests that at least a component of the effect may be mediated by a direct effect of REV-ERBα on expression of the *CLOCK* gene. REV-ERBα mediated regulation of *CLOCK* adds to the complexity of the feedback loop that maintains circadian rhythms. The BMAL1/CLOCK dimer is capable of up-regulating REV-ERBα, which will then repress *BMAL1* and *CLOCK* expression. This coordinate regulation of *BMAL1* and *CLOCK* by REV-ERBα may help maintain the circadian oscillations of various genes, including REV-ERBα itself (Figure 4c).
